# Nontuberculous Mycobacterial Infections: A Retrospective Analysis From a Tertiary Hospital in Portugal

**DOI:** 10.7759/cureus.74836

**Published:** 2024-11-30

**Authors:** Catarina La Cueva Couto, Maria Inês Ferreira, Clara Portugal, Elzara Aliyeva, Luís Carreto, Catarina Figueiredo Roquete, Fernando Rodrigues

**Affiliations:** 1 Pulmonology, Unidade Local de Saúde Amadora/Sintra, Amadora, PRT; 2 Pathology, Unidade Local de Saúde Amadora/Sintra, Amadora, PRT

**Keywords:** adverse effects, extra-pulmonary involvement, mycobacterium infections, nontuberculous mycobacteria, pulmonary involvement, treatment

## Abstract

Introduction

The prevalence of nontuberculous mycobacteria (NTM) is higher in patients with structural lung disease and in immunocompromised patients. Lung involvement is the most common. The *Mycobacterium avium* complex corresponds to the most identified agent. The treatment is complex and must be maintained for 12 months after cultural conversion. As it is a complex disease, the objective of this study is to carry out a demographic assessment of patients with NTM infection and evaluate the therapeutic regimens used and their adverse effects.

Materials and methods

A retrospective analysis of NTM isolates identified between 2012 and 2023 in a tertiary hospital in Portugal, focusing on those responsible for causing disease. The disease criteria were based on the guidelines established by the British Thoracic Society (BTS).

Results

We obtained a total of 35 patients with an average age of 63±15.5 years and the majority were male (n=22, 63%). Patients were divided into two groups: the group with lung disease (n=31, 89%); and the group with extra-pulmonary disease (n=4, 11%).

Within the group with lung disease, 15 (48%) had previous lung disease and 13 (42%) were immunosuppressed. The most common imaging pattern was nodular/bronchiectatic (n=25, 81%). The most isolated agents were *M. avium* (n=10, 32%), *M. intracelulare* (n=7, 23%) and *M. fortuitum *(n=4, 13%). The average treatment time was 13.5±3.8 months and the most used regimen was rifampicin, ethambutol and clarithromycin. During treatment, four (13%) presented hepatotoxicity, three (10%) nausea/vomiting and one (3%) ototoxicity. It was possible to identify nine (29%) susceptibility profiles, with only one (11%) patient showing resistance.

Within the group with extra-pulmonary involvement, all were immunosuppressed, three (75%) due to HIV infection. The affected organs were hepatic, lymph node, bone marrow and peritoneum. The isolated agents were *M. avium *(n=2, 50%), *M. kansasii *(n=1, 25%) and *M. triplex* (n=1, 25%). The average treatment time was 12±4.2 months. Two adverse effects were recorded: optic neuritis and nausea/vomiting. There is no data regarding the resistance profile.

Discussion

Pulmonary involvement was more prevalent and *M. avium* was the most predominant agent. Patients with pulmonary involvement had more underlying lung changes and those in the extra-pulmonary group had a greater degree of immunosuppression. The identification of NTM occurred mainly through cultural examination of sputum and bronchial secretions. The average duration of treatment was 13.5±3.8 months within the group with lung disease and 12±4.2 months within the group with extra-pulmonary involvement. The most documented adverse effects were nausea/vomiting and hepatotoxicity.

Conclusion

Our investigation intends to raise awareness of this pathology, which is a challenge in terms of treatment and diagnosis.

## Introduction

Nontuberculous mycobacteria (NTM) are species of mycobacteria other than *Mycobacterium tuberculosis* or *M. leprae* complex [1]. Around 200 NTM have been identified, and the number of new species has been increasing over the last few years [1]. NTM are ubiquitous organisms in the environment and can be isolated from water, soil, plants and animals [2,3]. Human-to-human transmission has not been documented [4].

Its prevalence is higher in patients with structural lung disease and in immunocompromised individuals [5]. Lung involvement is the most common in 80% of cases [6]. Extra-pulmonary involvement can affect multiple organs, including skin and soft tissues, ganglia and the musculoskeletal system [6]. *M. avium* complex (MAC) corresponds to the NTM agent most frequently identified as causing disease [7].

These bacteria can be categorized as slow, intermediate, or rapid growers based on their growth rate in culture. It is believed that humans contract the disease from environmental sources; when susceptible individuals inhale or ingest the bacteria, it can cause a chronic and progressive infection [8].

For the disease to be diagnosed, it is necessary to have compatible symptoms, imaging changes and microbiological isolations [9]. The decision to start treatment is not linear and must be based on the presence of disease criteria, risk of progression or dissemination and also the severity of the disease [7]. The risk of progression or dissemination is higher in patients with severe immunosuppression (particularly HIV infection), lung disease associated with cystic fibrosis, silicosis or lung transplantation, infection associated with exogenous material (central venous catheters (CVC), prostheses), chronic anemia, hypoalbuminemia, low BMI and advanced age [9]. The treatment of NTM disease is complex and prolonged and must be maintained for a minimum of 12 months after the negative culture test [7,9].

Therefore, with this work, we intend to carry out a demographic assessment of patients with infection by NTM identified in a tertiary hospital and evaluate the therapeutic regimens carried out and their respective adverse effects.

## Materials and methods

A retrospective analysis was carried out of all positive cultural cases for NTM in a tertiary hospital in Portugal, between 1999 and 2023, detected in any biological product, whether in inpatients or outpatients. Detailed isolate results were provided by the hospital’s Microbiology Unit. Patients who did not meet disease criteria for NTM according to British Thoracic Society (BTS) guidelines [8] and isolates from the period 1999 and 2011, due to lack of clinical data, were excluded.

A descriptive analysis of demographic and clinical data was obtained retrospectively through an analysis of patient records. Data analysis was conducted using the Statistical Package for the Social Sciences (IBM SPSS Statistics for Windows, IBM Corp., Version 20.0, Armonk, NY).

## Results

Of the 136 isolations obtained, isolations between 1999 and 2011 were excluded (n=74). Between January 2012 and December 2023, we obtained a total of 62 isolations of NTM, of which 35 were considered responsible for causing disease.

These patients had a mean age of 63±15.5 years (minimum age of 29 and maximum of 88), and the majority were male (n=22, 63%). Only five (14%) had a previous history of tuberculosis and seven (20%) had HIV infection. The variables analyzed divided by the two groups are summarized in Table 1.

**Table 1:** Variables analyzed by the two groups

Variables	Group with lung disease (n,%)	Group with extra-pulmonary disease (n, %)	Total (n, %)
Male gender	20 (65)	2 (50)	22 (63)
Previous history of tuberculosis	4 (13)	1 (25)	5 (14)
Previous lung disease	15 (48)	0	15 (43)
Immunosuppression	13 (42)	4 (100)	17 (49)
Symptoms			
Fever	11 (35)	2 (50)	13 (37)
Chronic cough	27 (87)	2 (50)	29 (83)
Sputum	25 (81)	1 (25)	26 (74)
Dyspnea	14 (45)	1 (25)	15 (43)
Hemoptysis	7 (23)	0	7 (20)
Night sweats	5 (16)	2 (50)	7 (20)
Weight loss	16 (52)	2 (50)	18 (51)
Fatigue	20 (65)	2 (50)	22 (63)
Radiographic features			
Nodular/bronchiectatic	25 (81)	2 (50)	27 (77)
Fibrocavitary	6 (19)	0	6 (17)
No lung changes	0	2 (50)	2 (6)
Isolations			
Mycobacterium avium	10 (32)	2 (50)	12 (34)
M. intracellulare	7 (23)	0	7 (20)
M. fortuitum	4 (13)	0	4 (10)
*M. abcessus* spp. *massiliense*	2 (7)	0	2 (6)
M. chelonae	2 (7)	0	2 (6)
M. genavense	2 (7)	0	2 (6)
M. kansasii	1 (3)	1 (25)	2 (6)
M. triplex	0	1 (25)	1 (3)
M. gordonae	1 (3)	0	1 (3)
M. lentiflavum	1 (3)	0	1 (3)
M. xenopi	1 (3)	0	1 (3)
Antibiotic susceptibility profile			
Polymerase chain reaction (PCR)	8 (26)	0	8 (23)
Antibiotic sensitivity testing	1 (3)	0	1 (3)
Absence	22 (71)	4 (100)	26 (74)
Outcome after treatment			
Death	6 (18)	1 (25)	7 (20)
Treated	20 (65)	3 (75)	24 (69)
Did not accept treatment	2 (7)	0	2 (6)
Under treatment	3 (10)	0	2 (6)
Adverse effects associated with treatment			
Optic neuritis	0	1 (25)	1 (3)
Nausea/vomiting	3 (10)	1 (25)	4 (11)
Hepatotoxicity	4 (12)	0	4 (11)
Ototoxicity	1 (3)	0	1 (3)

**Figure 1:**
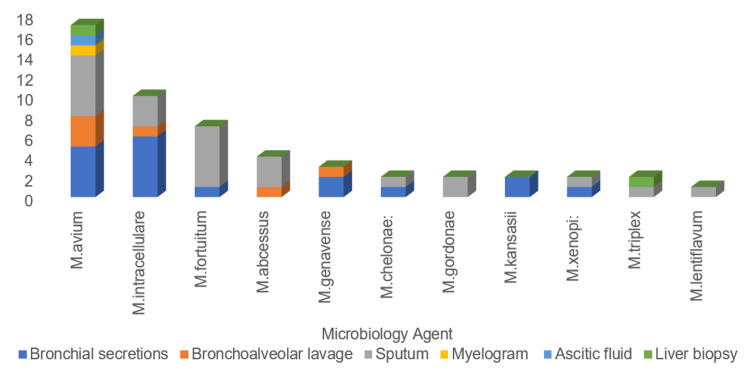
Biological products from which NTM isolates were obtained NTM: nontuberculous mycobacteria

An analysis was also carried out of the biological products in which each of these mycobacteria was identified (n=52) (sometimes there is more than one product per patient), which is represented in Figure 1. The identification of these mycobacteria occurred mainly in sputum (n=24, 46.2%) and bronchial secretions (n=18, 34.6%).

Group with lung disease

Within the group of patients with lung disease (n=31, 89%), a history of previous lung disease stands out in 15 (48%) of patients, including bronchiectasis (n=7, 23%), chronic obstructive pulmonary disease (COPD) (n=3, 10%), asthma (n=3, 10%), lung tumor (n=2, 6%), chronic pulmonary aspergillosis (n=1, 3%) and pulmonary fibrosis (n=1, 3%). It should also be noted that 13 (42%) had some degree of immunosuppression, four (13%) of which had HIV infection.

In terms of symptoms, more than half had a chronic cough (n=27, 87%), sputum (n=25, 81%), fatigue (n=20, 65%) and weight loss (n=16, 52%). In imaging, the most prevalent pattern was nodular/bronchiectatic (n=25, 81%). The most commonly isolated agents were* M. avium* (n=10, 32%), *M. intracellulare *(n=7, 23%) and *M. fortuitum* (n=4, 13%).

In terms of treatment, 20 (65%) of patients completed antibiotic therapy and six (18%) died during treatment. It should also be noted that two (7%) did not accept starting treatment and three (10%) are still undergoing it. Among patients who completed antibiotic therapy (n=20, 65%), the average duration of treatment was 13.5±3.8 months (minimum six months and maximum 20 months) and the most used regimen was rifampicin, ethambutol and clarithromycin (n=7, 35%). The schemes used are summarized in Table 2. Regarding the adverse effects presented, four (12%) presented hepatotoxicity, with two patients needing to suspend treatment after six months, three (10%) with nausea/vomiting and one (3%) with ototoxicity, with the need to suspend treatment after nine months.

**Table 2:** Therapeutic regimens used in the group of patients with pulmonary involvement * Strain resistant to clarithromycin, cefoxitin, ciprofloxacin, amoxicillin, linezolid, and doxycycline.

Therapeutic scheme carried out	n (%)	Duration of treatment	Agent (n)
Rifabutin, ethambutol, clarithromycin and streptomycin	1 (5)	9 months (suspended due to ototoxicity)	*Mycobacterium avium* (n=1)
Rifampicin, ethambutol and clarithromycin	7 (35)	Average of 13.3±1.4 months	*M. avium* (n=4) *M. intracellulare* (n=3)
Rifabutin, ethambutol and clarithromycin	2 (10)	Average of 6±0 months (suspended due to hepatotoxicity)	*M. avium* (n=2)
Levofloxacin and clarithromycin	1 (5)	15 months	*M. fortuitum* (n=1)
Linezolid, azithromycin and moxifloxacin	1 (5)	12 months	*M. chelonae* (n=1)
Amikacin, rifabutin, azithromycin, ethambutol and moxifloxacin	1 (5)	16 months	*M. xenopi *(n=1)
Ethambutol, clarithromycin and levofloxacin	1 (5)	12 months	*M. intracellulare* (n=1)
Rifabutin, ethambutol and azithromycin	2 (10)	Average of 13.5±2.1 months	*M. avium* (n=2)
Rifampicin, ethambutol and azithromycin	1 (5)	12 months	*M. genavense* (n=1)
Rifabutin, ethambutol and isoniazid	1 (5)	15 months	*M. kansasii *(n=1)
Azithromycin, moxifloxacin, clofazimine and ethambutol	1 (5)	18 months	*M. intracellulare *(n=1)
Amikacin, azithromycin, cefoxitin and clofazimine*	1 (5)	20 months	*M. abcessus *spp. *massiliense* (n=1)

From the point of view of the susceptibility profile to antibiotics, by polymerase chain reaction (PCR) or antibiotic sensitivity testing, summarized in Table 3, nine (29%) strains were studied, only one (11%) of which showed some type of resistance.

**Table 3:** Susceptibility profile and treatment carried out by identified species

Agent	Antibiotic susceptibility profile	Antibiotherapy performed
Mycobacterium avium	Search for gene resistance to macrolides and aminoglycosides: negative	Rifabutin, ethambutol, clarithromycin and streptomycin
M. avium	Search for gene resistance to macrolides and aminoglycosides: negative	Rifampicin, ethambutol and clarithromycin
M. avium	Search for gene resistance to macrolides and aminoglycosides: negative	Rifampicin, ethambutol and clarithromycin
M. avium	Search for gene resistance to macrolides and aminoglycosides: negative	Rifabutin, ethambutol and azithromycin
M. avium	Search for gene resistance to macrolides and aminoglycosides: negative	Rifampicin, ethambutol and clarithromycin
M. intracellulare	Search for gene resistance to macrolides and aminoglycosides: negative	Did not agree to start treatment
M. intracellulare	Search for gene resistance to macrolides and aminoglycosides: negative	Rifampicin, ethambutol and clarithromycin
M. intracellulare	Search for gene resistance to macrolides and aminoglycosides: negative	Azithromycin, moxifloxacin, clofazimine and ethambutol
*M. abscessus* C. (subsp. M. *massiliense*)	Strain sensitive to amikacin and resistant to clarithromycin, cefoxitin, ciprofloxacin, amoxicillin, linezolide, doxycycline	Amikacin, azithromycin, cefoxitin and clofazimine

Group with extra-pulmonary disease

Within the group of patients with extra-pulmonary infection (n=4, 11%), all had some degree of immunosuppression, with three (75%) having HIV infection and one (25%) having leukemia. Regarding the affected organs, the following stand out: hepatic (n=1, 25%); lymph node and lung (n=1, 25%); hepatic, node and lung (n=1, 25%); bone marrow and peritoneum (n=1, 25%). The most common symptoms, present in half of the patients (n=2), were fever, chronic cough, night sweats, weight loss and fatigue. Two (50%) of the patients had a nodular/bronchiectatic imaging pattern and two (50%) had no changes in the lung parenchyma. The most frequently isolated agents were *M. avium* (n=2, 50%), *M. kansasii* (n=1, 25%) and *M. triplex* (n=1, 25%).

Regarding the treatment carried out, one (25%) of the patients did not have a therapeutic regimen documented in the system and one (25%) died midway through treatment, which is why they were excluded from this sub-analysis. Thus, for the two (50%) patients in this group with complete treatment, the average treatment time was 12±4.2 months, with two different therapeutic regimens being used: rifabutin, ethambutol, moxifloxacin and azithromycin; isoniazid, ethambutol and azithromycin. In this subgroup, two adverse effects were recorded: optic neuritis associated with ethambutol and persistent nausea/vomiting. Please note that there is no data regarding the resistance profile of these four identified agents.

## Discussion

The term NTM refers to species of mycobacteria other than the *M. tuberculosis* complex and the mycobacteria responsible for causing leprosy [1]. The prevalence of NTM has increased over the years, making its diagnosis and treatment challenging. They mostly cause lung disease, mainly affecting middle-aged people, as observed in our study. Males have the highest incidence worldwide, as is the case in our sample, especially in NTM infection with pulmonary involvement [3,10]. The overall relative prevalence of NTM lung disease is estimated to be around 90%, which is similar to our data results [11].

The risk factors for disease with pulmonary and extra-pulmonary involvement are different. Older age and lung structural changes are more associated with lung-affecting disease [3,6]. Immunosuppression, namely HIV infection, is more associated with extra-pulmonary involvement, as observed in our results, with all patients in the extra-pulmonary group presenting some degree of immunosuppression [3,6].

The imaging pattern in lung disease caused by NTM can be of two types: the fibrocavitary pattern, which is more associated with men who smoke, and the nodular/bronchiectatic pattern, which is more associated with women without structural lung changes [4]. Therefore, the prevalence of imaging changes differs depending on the most prevalent sex in a given region, and in Europe, the most common pattern is fibrocavitary [6]. However, given that the most prevalent sex in our study was male, it is understood that the most prevalent pattern was nodular bronchiectatic, which is in agreement with the literature.

The NTM species most responsible for causing lung disease are MAC and *M. fortuitum*, as observed in our patients: MAC (which includes *M. avium* and *M. intracellulare*) is responsible for 17 (55%) patients and *M. fortuitum *for four (13%) [3,4]. The species most responsible for causing extra-pulmonary disease described in the literature are MAC, *M. kansasii* and *M. abscessus*, and the first two species were also among the most identified in our study, with two (50%) and one (25%) respectively, despite our sample being small (four strains) [3,4].

From a treatment point of view, this depends on the species of NTM identified, as well as the respective resistance. As recommended in the treatment of tuberculosis, this infection should be treated with a regimen of multiple antibiotics, which is why susceptibility to these antibiotics should always be tested [3]. According to the guidelines of the American Thoracic Society/Infectious Diseases Society of America (ATS/IDSA) and BTS, macrolides are the basis of treatment, especially in MAC agents, which is why the identification of resistance to this antibiotic is the main predictor of failure therapeutic [4,6]. Treatment must be maintained for a minimum of 12 months after crop conversion. Clinical and imaging improvements must also be achieved to consider therapeutic success [4,6]. In this series, everyone was treated with at least two antibiotics, with an average treatment period of approximately 13.5±3.8 months within the group with lung disease and 12±4.2 months within the group with extra-pulmonary involvement, as recommended. Highlighting three patients who had to suspend treatment at six and nine months due to serious adverse effects, such as hepatotoxicity and ototoxicity. It should also be noted that, of the nine available susceptibility profiles, all MAC strains were sensitive to macrolides and only the *M. abscessus* strain showed multiple resistance, including to a macrolide (clarithromycin).

Although the sample is small, given the rarity of this disease, it seemed pertinent to carry out this work to raise awareness of this pathology, which is still a challenge in terms of treatment and diagnosis. The main limitations of this series are the limited data on susceptibilities to antibiotics of different strains, as well as the almost absence of requests for cultures after the start of therapy, making it impossible to control the effectiveness of the treatment (partly because these patients are subsequently monitored in the Centers for Pulmonological Diagnosis and there is no data in the system). Therefore, in the future, with the advancement of new genetic technologies, we hope that laboratory studies will be more complete and rapid, like tissue biopsy and culture for extrapulmonary cases or nucleic acid amplification tests for species identification, allowing for faster diagnosis and targeted treatment.

## Conclusions

In conclusion, an analysis of the demographic, clinical, imaging and microbiological data of the NTM responsible for causing disease over 12 years in a tertiary hospital in Portugal was carried out. Lung involvement was the most common, particularly for those with underlying lung disease or weakened immune systems. Mycobacteria belonging to MAC were the most prevalent, more specifically *M. avium*, as described in the literature. Patients with pulmonary involvement had more structural lung changes and those in the extra-pulmonary group had a greater degree of immunosuppression. The identification of NTM occurred mainly through cultural examination of sputum and bronchial secretions. Treatment regimens depend on the species of NTM, site of infection, severity, and individual patient factors. The average duration of treatment was similar to that found in the literature, around 13 months, despite the different therapeutic regimens used. The most documented adverse effects were nausea/vomiting and hepatotoxicity. With this series, we intend to raise awareness of this pathology, which is a challenge in terms of treatment and diagnosis.
